# Efficacy and safety of L-ornithine L-aspartate combined with lactulose in treatment of hepatic encephalopathy: a systematic review and meta-analysis of randomized controlled trial

**DOI:** 10.3389/fmed.2025.1581792

**Published:** 2025-04-30

**Authors:** Hui Zhang, Yujuan Fu, Minghao Lin, Zheng Nan, Dexi Zhao

**Affiliations:** ^1^Changchun University of Chinese Medicine, Changchun, China; ^2^The Affiliated Hospital to Changchun University of Chinese Medicine, Changchun, China

**Keywords:** hepatic encephalopathy, l-ornithine l-aspartate, lactulose, meta-analysis, randomized controlled trial

## Abstract

**Background:**

Hepatic encephalopathy (HE) represents a collection of metabolic disturbances and regulatory imbalances within the central nervous system that result from advanced liver conditions.

**Purpose:**

This article explores the efficacy and safety evaluation of L-ornithine L-aspartate (LOLA) combined with lactulose in the treatment of hepatic encephalopathy based on meta-analysis, providing a rational reference for clinical medication use.

**Methods:**

Following the PICOS principle, we searched for literature on the treatment of hepatic encephalopathy with Ornithine aspartate combined with lactulose. The literature search was conducted up to and including September 21, 2024. For studies that met the criteria, the Review Manager 5.4 software was used to perform a meta-analysis.

**Results:**

A total of 12 articles were ultimately included, involving 858 patients, with 433 in the treatment group and 425 in the control group. Meta-analysis results: In terms of the total effective rate (RR: 1.31, 95%CI: 1.22, 1.42), the result is statistically significant (*Z* = 7.15, *P* = 0.00001 < 0.05), and for AST, ALT, NH3, TBIL, *P* = 0.00001 < 0.05. The pooled (RR: 1.31 [95% CI = 1.22, 1.42]), which is statistically significant, LOLA and Lactulose is 31% more effective than Lactulose alone in the control group in treating HE.

**Conclusion:**

This study indicates that the combination of LOLA and lactulose in the treatment of hepatic encephalopathy has a higher total effective rate in clinical practice and can significantly reduce the levels of AST, ALT, TBIL, and NH3.

**Systematic review registration:**

https://www.crd.york.ac.uk/PROSPERO/, identifier CRD42024592957.

## 1 Introduction

Hepatic Encephalopathy (HE) is a disorder of central nervous system dysfunction induced by metabolic disturbances resulting from severe liver disease (1). Hepatic encephalopathy is often triggered by severe viral hepatitis and severe toxic hepatitis, or by the progression of drug-induced or alcoholic cirrhosis to the end stage of diffuse liver disease, with approximately 70% of all cases of hepatic encephalopathy caused by end-stage cirrhosis (2). Patients with hepatic encephalopathy can be classified into two main categories: overt hepatic encephalopathy and covert hepatic encephalopathy. In the early stages of the disease, patients often exhibit subtle symptoms, making it difficult to detect significant abnormal clinical signs. At this time, patients may show behavioral changes, disturbances in mental status, and even coma. However, subtle abnormal symptoms can be detected through psychological assessments and neurophysiological testing, which can aid in early detection and diagnosis (3). Although the specific pathogenesis of HE is not yet fully understood, the ammonia hypothesis is widely recognized among the various theories related to it. According to the ammonia hypothesis, when liver function is severely decompensated, a large amount of ammonia cannot be normally metabolized by the liver and instead directly enters the systemic circulation, leading to hyperammonemia. In this process, ammonia triggers abnormal reactions in astrocytes, promoting the conversion of glutamate to glutamine in the brain, which subsequently inhibits the normal function of the central nervous system and causes dysregulation. Concurrently, this process leads to a compensatory decrease in osmolytes. When the capacity regulation function of astrocytes becomes uncompensated, it may trigger cerebral edema, further leading to increased intracranial pressure, and in severe cases, posing a threat to life. According to data from the World Health Organization (WHO), the incidence of HE globally ranges between 10 and 50%, showing significant regional differences, with up to 40% of hospitalized patients with cirrhosis suffering from minimal hepatic encephalopathy. If such diseases are not treated promptly and effectively in the early stages, patients are prone to progress to a state of metabolic, circulatory, and neurological system dysfunction, often leaving sequelae and irreversible functional damage. In clinical practice, elevated serum ammonia levels are considered one of the key factors in the pathogenesis of hepatic encephalopathy, making the reduction of serum ammonia levels a primary task in clinical treatment. Common therapeutic measures include correcting electrolyte disorders, regulating acid-base imbalances, and reducing serum ammonia concentrations. These methods can temporarily alleviate the clinical symptoms of hepatic encephalopathy but are extremely difficult to fundamentally block the production of blood ammonia and prevent damage to the central nervous system. This article focuses on exploring the clinical efficacy and safety of LOLA combined with lactulose in the treatment of hepatic encephalopathy.

## 2 Materials and methods

This study has been registered with the PROSPERO (International Prospective Register of Systematic Reviews) under the registration number CRD42024592957. This Meta-analysis study is conducted strictly in accordance with the guidelines of the Preferred Reporting Items for Systematic Reviews and Meta-Analyses (PRISMA). As is shown in PRISMA_2020_checklist. As of September 20, 2024, searches were conducted in Pubmed, Cochrane Library, Web of Science, Embase, CBM, CNKI, VIP, and Wanfang Database. PubMed Specific search strategies are shown in Supplementary Material 1. There are no language restrictions. The reference lists of the identified literature were manually reviewed to find potential eligible studies.

### 2.1 Inclusion criteria

#### 2.1.1 Type of study

Publicly published randomized controlled trials (RCTs) are included in this study, with no restrictions on language, country, publication date, or stage.

#### 2.1.2 Study subjects

Patients diagnosed with HE can refer to the diagnostic criteria for cirrhosis with hepatic encephalopathy established by the American Association for the Study of Liver Diseases (AASLD) and the European Association for the Study of the Liver (EASL), which may also have published relevant guidelines for the diagnosis and treatment of hepatic encephalopathy. All included cases were diagnosed with hepatic encephalopathy. This study includes patients aged 18-80, with no gender restrictions, and no limitations on race, disease course, or disease severity.

#### 2.1.3 Interventions

Treatment Group: In addition to the control group’s regimen, Ornithine aspartate injection was administered intravenously at a dose of 10 g/d, once per day.

Control Group: Lactulose treatment was administered orally at a dose of 10 mL per dose or by enema at 100 mL, three times per day.

#### 2.1.4 Outcome indicators

Total effective rate, adverse reactions, ALT, AST, TBIL, NH3.

### 2.2 Exclusion criteria

RCTs will be excluded if they meet any of the following conditions: ① Do not meet the standards for clinical efficacy evaluation; ② Duplicate reports; ③ Interventions involving traditional Chinese medicine, acupuncture, or combined treatment with other drugs; ④ Lack of relevant outcomes; ⑤ Inability to obtain the full text of the study; ⑥ Incorrect or incomplete data.

### 2.3 Search strategy

A computer search was conducted for published studies related to the treatment of HE with LOLA and lactulose. Databases searched include PubMed, Cochrane Library, Embase, Web of Science, CBM, CNKI, WANFANG DATA, and VIP. Search terms included: Hepatic Encephalopathy, Encephalopathies, Hepatic, Hepatic Encephalopathies, Portosystemic Encephalopathy, Ornithylaspartate, Orn-Asp, Lactulose, etc. The search covered the period from the inception of each database up to September 21, 2024, with no restrictions on publication year or language. Searches were conducted using a combination of Medical Subject Headings (MeSH) terms and free-text words.

### 2.4 Data extraction

All articles were managed using Endnote software by two individuals (Hui Zhang and Minghao Lin), with any disagreements resolved through discussion with a third party, Dexi Zhao. Selection was made by excluding irrelevant articles, reviews, and animal experiments. Two evaluators independently extracted data from eligible studies using Excel (Microsoft, United States), and the collected information included the following: sample size, gender, and age for each group; intervention measures; outcome indicators: ① Total effective rate; ② Adverse reactions; ③ NH3; ④ Alanine aminotransferase (ALT); ⑤ Aspartate aminotransferase (AST).

### 2.5 Literature quality assessment

We used the Cochrane Risk of Bias assessment tool to evaluate the methodological quality of the included studies. In the quality methodology assessment process, we will focus on examining the following criteria: whether the generation of the random sequence is appropriate; whether allocation concealment measures were implemented; whether subjects and intervention providers were blinded; whether outcome assessors were also masked; the completeness of the outcome data; the existence of selective reporting of results; and whether there are other potential sources of bias. Based on the relevant assessment criteria, included studies will be rated as “low risk,” “high risk,” or “unclear risk.” In case of disagreements on the quality assessment, a third researcher will be consulted.

### 2.6 Heterogeneity assessment

I^2^ and Q test were utilized to evaluate the consistency of the included studies. Generally, an I^2^ ≤ 50% and a *Q*-test *P* ≥ 0.1 suggest low heterogeneity, prompting the use of a fixed-effects model for analysis. On the other hand, an I^2^ > 50% and a *Q*-test *P* < 0.1 to significant heterogeneity, advocating for a random-effects model. When substantial heterogeneity is identified, sensitivity or subgroup analyses are conducted to investigate potential causes. Sensitivity analysis involves the sequential exclusion of individual studies. Changes in heterogeneity following the removal of certain studies may signal their contribution to the inconsistency, prompting further investigation into their impact. If heterogeneity persist despite study exclusions, it suggests that the findings are stable and reliable.

### 2.7 Statistical methods

The Meta-analysis is performed using Revman 5.4 provided by the Cochrane Collaboration. When the outcome indicator is a binary variable, the Risk Ratio (RR) is used as the effect size. When the outcome indicator is a continuous variable, the weighted mean difference (MD) is used as the effect size, and all effect sizes should be expressed with a 95% confidence interval (CI).

### 2.8 Publication bias

When analyzing the effect size, if the number of included articles exceeds 10, a funnel plot can be used to assess the risk of publication bias. A noticeably asymmetric funnel plot indicates the presence of publication bias.

## 3 Meta-analysis results

### 3.1 Literature search

**FIGURE 1:**
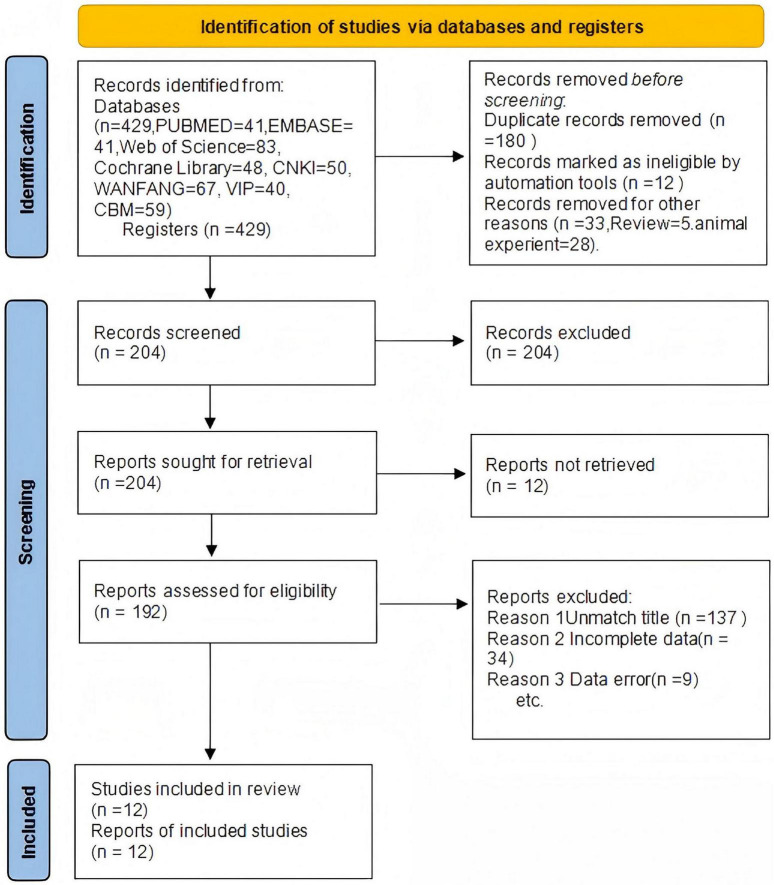
Flow diagram.

**FIGURE 2:**
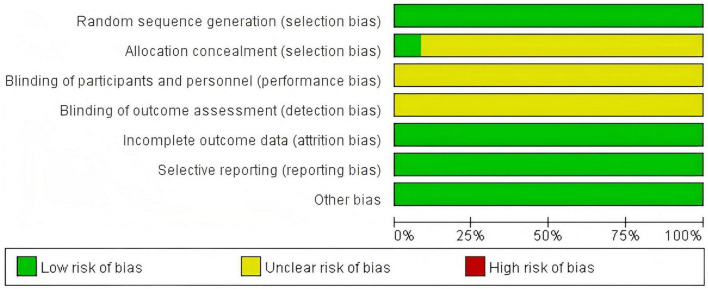
Risk of bias graph.

**FIGURE 3:**
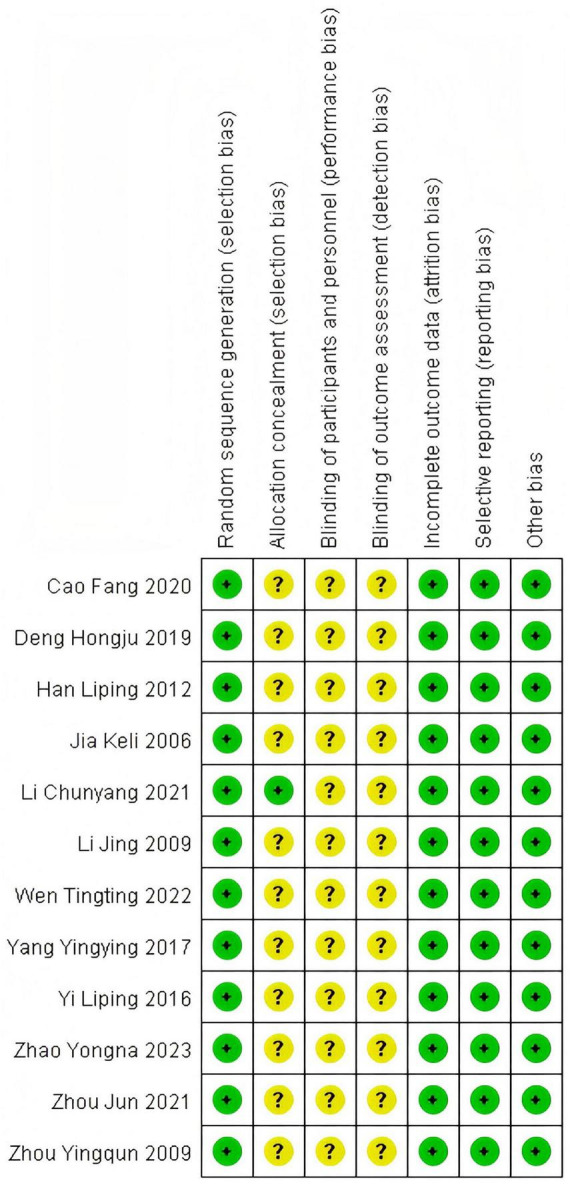
Risk of bias summary.

A total of 12 (4–15) articles were ultimately included, with 433 cases in the treatment group and 425 cases in the control group, totaling 858 patients. The drugs included in this article are as follows: LOLA combined with Lactulose. The Flow diagram is shown in Figure 1, and the basic information of the included literature is presented in Table 1. Twelve articles were considered to be at low risk of bias and of high quality, with the met criteria marked as “+” and the unmet criteria as “-” in the figure. Figures 2, 3 is a statistical chart of the proportion of methodological assessment items. Due to different drugs and prices, and no mention of blinding, they are all colored yellow.

**TABLE 1:** Basic information.

ID	Author	Sample size		Sex(M/F)		Age (ẋ ± s)		Time (ẋ ± s) year		Intervention measure		Therapy time
		T	C	T	C	T	C	T	C	T	C	
1	Zhou and Shen (5)	37	37	32/5	33/4	48.31 ± 3.12	49.28 ± 3.15	5.17 ± 0.36	5.20 ± 0.35	OA+LC	LC	1 w
2	Yi (6)	46	39	38/8	33/9	−	−	−	−	OA+LC	LC	1 w
3	Li (8)	30	30	17/13	16/14	42.20 ± 5.10	42.50 ± 5.30	−	−	OA+LC	LC	2 w
4	Wen and Xu (9)	43	43	24/19	26/17	52.51 ± 5.43	52.68 ± 5.47	7.06 ± 1.26	6.89 ± 1.32	OA+LC	LC	2 w
5	Li (10)	43	43	−	−	−	−	−	−	OA+LC	LC	1 w
6	Han (7)	30	30	−	−	−	−	−	−	OA+LC	LC	1 w
7	Deng et al. (11)	60	60	35/25	38/22	53.38 ± 6.86	52.65 ± 6.29	6.31 ± 2.09	6.91 ± 2.27	OA+LC	LC	2 w
8	Cao et al. (12)	47	47	28/19	30/17	53.74 ± 5.70	53.70 ± 5.61	5.83 ± 1.13	5.78 ± 1.09	OA+LC	LC	2 w
9	Zhao et al. (13)	20	20	11/9	12/8	52.97 ± 1.65	53.08 ± 4.17	4.89 ± 9.04	5.68 ± 6.57	OA+LC	LC	2 w
10	Jia (14)	28	27	20/8	18/9	−	−	−	−	OA+LC	LC	1 w
11	Zhou (15)	26	26	18/8	19/7	51.45 ± 6.27	50.20 ± 5.79	−	−	OA+LC	LC	4 w
12	Yang (4)	23	23	15/8	17/6	51.9 ± 7.2	52.2 ± 8.3	6.7 ± 1.1	7.0 ± 0.9	OA+LC	LC	2 w

### 3.2 Meta-analysis

#### 3.2.1 Efficacy rate

##### 3.2.1.1 Heterogeneity test

**FIGURE 4:**
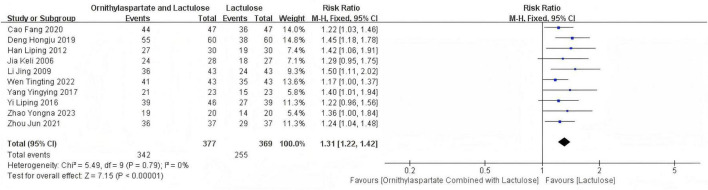
Forest plot of efficacy rate.

In this study, 10 articles were subjected to heterogeneity testing, with I^2^ = 0% and Q test *P* = 0.79 > 0.1. The pooled (RR:1.31 [95% CI = 1.22, 1.42]), which is statistically significant, *Z* = 7.15, *P* = 0.00001 < 0.05, indicating that the combination therapy of LOLA and Lactulose is more effective in treating hepatic encephalopathy (HE) than Lactulose alone in the control group, as is shown in Figure 4.

##### 3.2.1.2 Bias test

**FIGURE 5:**
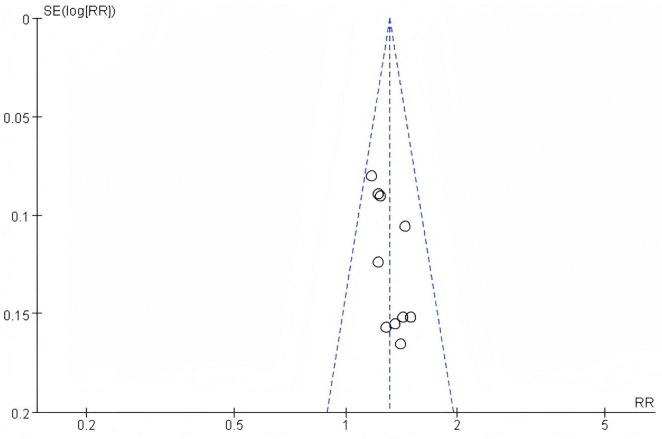
Funnel plot of efficacy rate.

By drawing a funnel plot, we examine whether there is publication bias in this study. The funnel plot for this study is as follows: It can be clearly seen that there is no publication bias in the literature of this study, and the funnel plot is symmetrical, as is shown in Figure 5.

#### 3.2.2 Adverse reactions

##### 3.2.2.1 Heterogeneity test

**FIGURE 6:**
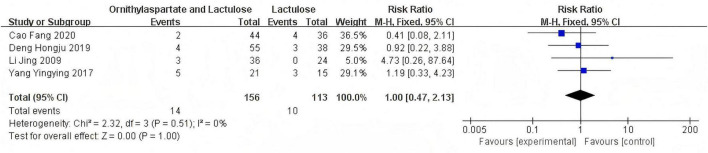
Forest plot of adverse reactions.

In this study, 4 articles were subjected to heterogeneity testing, with I^2^ = 0% and the *P* = 0.51 > 0.1, (RR: 1.00 [95%CI = 0.47, 2.13]), which is not statistically significant, *Z* = 0.00, *P* = 1.00 > 0.05. The adverse reactions reported in the included articles are specifically manifested in the gastrointestinal and cardiac system, as is shown in Figure 6.

#### 3.2.3 Neurotoxin substance ammonia NH3 (μmol/L)

##### 3.2.3.1 Heterogeneity test

**FIGURE 7:**
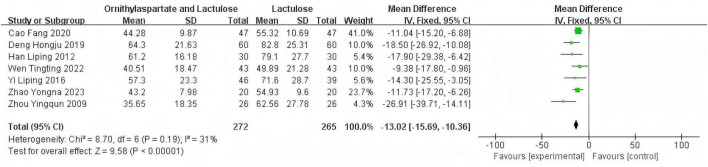
Forest plot of NH3.

In this study, 11 articles were assessed for NH3. Heterogeneity testing showed I^2^ = 93% > 50%, and the *Q*-test *P* < 0.01. Sensitivity analysis of the 11 articles revealed that the studies by Jia (14), Yingying (4), Li (8), and Zhou and Shen (5) significantly influenced the heterogeneity. After excluding these studies, heterogeneity testing showed that the remaining 7 articles had lower heterogeneity (I^2^ = 31% < 50%, *p* = 0.19 > 0.1), and a fixed-effect model was used for the Meta-analysis. RR: −13.02 (95%CI [−15.69, −10.36]), which is statistically significant. *Z* = 9.58, *P* < 0.00001, suggesting that LOLA combined with Lactulose is more effective in restoring the levels of the neurotoxin substance in patients with HE, as is shown in Figure 7.

#### 3.2.4 Aspertate aminotransferase

##### 3.2.4.1 Heterogeneity test

**FIGURE 8:**
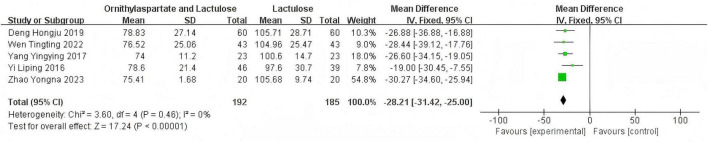
Forest plot of AST.

In this study, 8 articles were assessed for AST. Heterogeneity testing showed I^2^ = 96% > 50%, and the *Q*-test *p* < 0.01. Sensitivity analysis of the 8 articles revealed that the studies by Zhou and Shen (5), Li (8), and Han (7) significantly influenced the heterogeneity. After excluding these studies, heterogeneity testing showed that the remaining 5 articles had lower heterogeneity (I^2^ = 0% < 50%, *p* = 0.46 > 0.1), and a fixed-effect model was used for the Meta-analysis. (RR: −28.21, [95%CI: −31.42, −25]), which is statistically significant. *Z* = 17.24, *P* < 0.00001, suggesting that LOLA combined with Lactulose is more effective in restoring liver function in patients with HE, as is shown in Figure 8.

#### 3.2.5 Alanine transaminase

##### 3.2.5.1 Heterogeneity test

**FIGURE 9:**
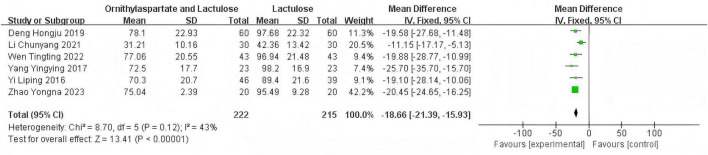
Forest plot of ALT.

In this study, 9 articles were assessed for AST. Heterogeneity testing showed I^2^ = 90% > 50%, and the *Q*-test *P* < 0.01, indicating that the selected literature has statistically significant heterogeneity, necessitating a search for heterogeneity. Sensitivity analysis of the 9 articles revealed that the studies by Zhou and Shen (5), Jia (14), and Han (7) significantly influenced the heterogeneity. After excluding these studies, heterogeneity testing showed that the remaining 6 articles had lower heterogeneity (I^2^ = 43% < 50%, *P* = 0.12 > 0.1), and a fixed-effect model was used for the Meta-analysis. (RR: −18.66 [95%CI: −21.39, −15.93]), which is statistically significant. *Z* = 13.41, *P* < 0.00001, suggesting that LOLA combined with Lactulose is more effective in restoring liver function in patients with HE, as is shown in Figure 9.

#### 3.2.6 Total bilirubin

##### 3.2.6.1 Heterogeneity test

**FIGURE 10:**
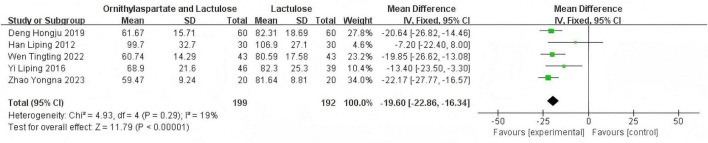
Forest plot of TBIL.

In this study, seven articles were assessed for TBIL. In the heterogeneity test, the I^2^ value was 70%, which exceeded the threshold of 50%. Meanwhile, the *p*-value of the Q test was less than 0.01, indicating that there was statistically significant heterogeneity among the 7 articles included in this study. Therefore, it was necessary to further investigate the sources of heterogeneity. Through sensitivity analysis of the 7 articles, it was found that the studies by Jia (14) and Yingying (4) had a significant impact on the heterogeneity. After excluding these two studies, we conducted the heterogeneity test again. The results showed that the heterogeneity among the remaining five articles was significantly reduced, I^2^ = 19%, *P* = 0.29<0.05, which was greater than the significance level of 0.1. Based on this result, a fixed-effect model was used for the Meta-analysis. By synthesizing the data of 6 studies, including the two previously excluded ones (although they were excluded in the heterogeneity analysis, they were still included in the calculation of the RR value here to maintain the integrity of the summary data, but in the actual analysis, only the remaining 5 articles should be used), (RR: −19.60, [95% CI: −22.86, −16.34]). This result was statistically significant (*Z* = 11.79, *P* < 0.00001), indicating that the combination of Ornithine aspartate and lactulose for the treatment of hepatic encephalopathy resulted in better liver function recovery, as is shown in Figure 10.

##### 3.2.6.2 Bias test

**FIGURE 11:**
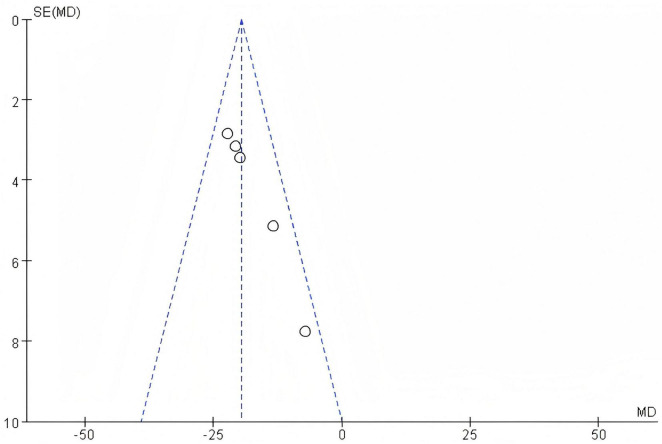
Funnel plot of TBIL.

The funnel plot of this study, as shown in the following figure, is essentially symmetrical. Therefore, it can be concluded that there is no publication bias in the literature of this study, as is shown in Figure 11.

## 4 Discussion

Previous meta-analyses have demonstrated that LOLA is significantly superior to placebo or conventional treatment in improving neuropsychiatric symptoms, blood ammonia levels, and overall response rates in patients with HE (16). The strengths of our current study lie in its RCT design, which includes a significantly larger number of patients and a greater diversity of etiologies compared to previous studies, thereby further validating the efficacy and safety of LOLA. In the current field of medical research, LOLA has become a research subject that has attracted much attention. Meta-analyses on it are not absent either, and many scholars have carried out explorations from their respective perspectives. This study has unique advantages. One of the most prominent advantages is that, during the research process, an innovative optimization has been made in the literature search process—expanding the time range to conduct a more comprehensive search. However, despite the broad clinical application prospects of ornithine aspartate, there is still a lack of systematic evaluation on the therapeutic regimen combining ornithine aspartate with lactulose. Combination therapy has demonstrated significant advantages in improving liver function indicators in patients. Compared with monotherapy using lactulose, combination therapy can reduce NH3 levels by 13%, AST by 28%, ALT by 19%, and TBIL by 20%. The reduction in ammonia levels can significantly alleviate patients’ neurological symptoms, such as impaired consciousness and abnormal behavior, thereby improving their quality of life and cognitive function and reducing the risk of recurrence of hepatic encephalopathy. AST is one of the important indicators reflecting liver injury. Its reduction indicates that combination therapy can more effectively alleviate liver inflammation and injury, which is conducive to the repair and regeneration of liver cells and thus improves overall liver function. ALT is also an important indicator for assessing liver injury. The significant reduction in ALT levels due to combination therapy suggests that it has a significant advantage in protecting liver cells and reducing hepatocyte necrosis, which helps maintain the normal metabolic function of the liver. The decrease in TBIL indicates that combination therapy can more effectively improve the liver’s detoxification and bile secretion functions, reduce the accumulation of bilirubin in the body, lower the incidence and severity of jaundice, and improve patients’ symptoms of skin and scleral icterus, thereby alleviating their discomfort. Overall, the significant improvements in multiple key liver function indicators with combination therapy indicate that it has a more comprehensive and effective advantage in treating liver diseases, which can bring better prognosis and quality of life improvement for patients. In existing clinical practice, the combined use of ornithine aspartate and lactulose has proven to exhibit significant efficacy in the treatment of hepatic encephalopathy. As a disaccharide, lactulose enters the body and is broken down into lactic acid by bacteria, which can lower the intestinal pH value, thereby reducing the formation of ammonium salts, decreasing the body’s absorption of ammonia, and subsequently lowering blood ammonia levels. When lactulose is used in combination with ornithine aspartate, their synergistic effect can significantly decrease blood ammonia levels, promote liver function recovery, effectively alleviate hepatic encephalopathy and cerebral edema, and improve patients’ neurological function. Although some studies have reported the positive effects of ornithine aspartate combined with lactulose in treating hepatic encephalopathy, these studies are mostly small-sample clinical observations or case analyses, lacking validation from large-scale, multicenter, randomized controlled trials. Therefore, the systematic evaluation of this combined therapeutic regimen remains inadequate and requires further exploration (3, 16). Based on previous research, this paper expands the literature review, aiming to comprehensively explore the clinical application effects of ornithine aspartate combined with lactulose. Through systematic evaluation of the efficacy, safety, and economy of this combined therapeutic regimen, it can provide a more reliable basis for clinical decision-making and also contribute to the development and optimization of related drugs. In the future, it is anticipated that more high-quality studies will be included to jointly promote the widespread application and development of the ornithine aspartate and lactulose combined therapeutic regimen in clinical practice.

Hepatic encephalopathy is a common and severe complication in patients with liver disease, with a complex pathogenesis involving multiple factors such as ammonia toxicity and neurotransmitter disturbances. Traditional treatment methods have certain limitations. Ornithine aspartate and lactulose each play a role in the treatment of hepatic encephalopathy, and their combined application may offer unique advantages, bringing hope for improving the prognosis of patients with this condition. The combination therapy primarily exhibits significant advantages in ammonia metabolism and liver function improvement. Firstly, in terms of ammonia metabolism, ornithine aspartate mainly promotes the metabolism and conversion of ammonia to urea in the liver, thereby reducing the accumulation of ammonia in the body from the source. Meanwhile, lactulose reduces ammonia production and promotes ammonia excretion in the intestine. Together, they form a comprehensive ammonia metabolism regulation system from the liver to the intestine, enabling more efficient reduction of blood ammonia levels. This synergistic effect helps rapidly correct hyperammonemia in patients with hepatic encephalopathy, thereby alleviating neuropsychiatric symptoms caused by ammonia toxicity. In terms of hepatocyte improvement, ornithine aspartate’s role in hepatocyte repair and regeneration, combined with lactulose’s effect of reducing liver ammonia load, contributes to the overall improvement of liver function. As liver function gradually recovers, its detoxification, synthesis, and other functions are enhanced, enabling better management of issues such as toxin accumulation in the body. The combined application of these two drugs can regulate protein metabolism and energy metabolism in the liver, maintain the stability of the intrahepatic environment, facilitate the normal function of hepatocytes, and fundamentally improve the pathophysiological state of hepatic encephalopathy. The combination of ornithine aspartate and lactulose for the treatment of hepatic encephalopathy demonstrates significant superiority. From the perspective of mechanism of action, they have a synergistic effect, more effectively regulating ammonia metabolism and improving liver function. In terms of clinical efficacy, compared with traditional treatment methods, this combination can more rapidly reduce blood ammonia, improve neuropsychiatric symptoms, and reduce recurrence rates. In terms of safety, it does not significantly increase the risk of adverse reactions. Therefore, this combined therapeutic regimen has broad application prospects in the treatment of hepatic encephalopathy and is worthy of further promotion and in-depth research in clinical practice.

This article discusses the treatment of hepatic encephalopathy with LOLA combined with lactulose. When liver function in patients with cirrhosis becomes abnormal, it leads to the establishment of portal vein collateral circulation, allowing ammonia to enter brain tissue through these collaterals and subsequently cause central nervous system dysfunction. Lactulose can reduce the acidity in the intestines, which helps to expel ammonia from the blood-brain barrier, thereby reducing its toxic damage to the central nervous system. On the other hand, LOLA can directly affect enzyme activity in the liver, effectively eliminate a large number of free radicals, and reduce blood ammonia levels (17). The following literature compares the combination therapy with LOLA. The total effective rate of the combination therapy (88.89%) was significantly higher than that of the LOLA group (66.67%), with a statistically significant difference (*P* < 0.05). The combination of LOLA and lactulose showed significant therapeutic effects in patients with HE. It not only improved liver function and reduced blood ammonia levels, but also enhanced cognitive function in patients (18). When comparing combination therapy with lactulose, the total clinical effective rate of the observation group (95.0%) was higher than that of the control group (70%), with a statistically significant difference between groups (*P* < 0.05) (13). The combination of LOLA and lactulose oral solution for the treatment of hepatic encephalopathy demonstrated significant clinical efficacy, reducing the levels of inflammatory factors and neurotoxic substances. The combined use of LOLA and lactulose as a treatment plan can significantly improve the intestinal microenvironment in patients with hepatic encephalopathy, effectively blocking the synthesis and absorption of exogenous toxic ammonia (19). This combined treatment approach not only promotes the recovery of liver function but also alleviates the symptoms of hepatic encephalopathy and cerebral edema, thereby improving patients’ neurological functions and effectively enhancing their quality of life. LOLA was included in the German Pharmacopoeia in 1991, and subsequently, the United States. Food and Drug Administration (FDA) approved specific drugs for the treatment of alcohol withdrawal and hepatic encephalopathy (20). In the “Practice Guidelines for Hepatic Encephalopathy Due to Chronic Hepatitis” by the American Association for the Study of Liver Diseases and the European Association for the Study of the Liver, LOLA is listed as the preferred treatment for hepatic encephalopathy. LOLA, as a complex of L-ornithine and L-aspartate, is an indispensable substrate for the synthesis of urea and glutamine (21). Within hepatocytes, aspartate is involved in the synthesis of nucleic acids and glutamine, producing oxaloacetic acid, which supplies energy to tissue cells through the tricarboxylic acid cycle and the urea cycle, achieving the dual detoxification function of the liver, protecting and promoting the uptake, transformation, and self-repair and regeneration of hepatocytes, and rapidly reducing blood ammonia concentrations. Ornithine, as an activator of the urea cycle, not only participates in the activation process but also combines with ammonia in the body circulation, promoting urea synthesis, accelerating the excretion of ammonia, and enhancing detoxification effects. Lactulose, an artificially synthesized non-absorbable disaccharide, is mainly broken down into lactic acid and acetic acid in the right colon, significantly reducing the colonic pH value, reducing the production of ammonia, amines, and thiols, converting NH3 into NH4+, inhibiting the growth of alkaline bacteria in the intestine, and thus reducing the endotoxins produced by protein breakdown.

In this study, the combined treatment of LOLA and lactulose for hepatic encephalopathy not only effectively reduced patients’ blood ammonia levels but also demonstrated significant efficacy in restoring hepatocyte function. The adverse reactions reported in the literature included in this study were mostly concentrated in the gastrointestinal and cardiocerebrovascular systems. LOLA is a hypertonic solution. Rapid infusion or high oral doses can increase intestinal osmotic pressure, stimulate intestinal peristalsis, and lead to diarrhea and abdominal pain. Aspartate and ornithine participate in the urea cycle, which may indirectly affect intestinal pH and irritate the gastrointestinal mucosa. High doses may consume blood ammonia through the urea cycle, leading to hypokalemia or hyponatremia, potentially triggering arrhythmias or blood pressure fluctuations. Ornithine metabolism may produce nitric oxide (NO), causing vasodilation and resulting in transient hypotension or dizziness. Lactulose is broken down by colonic bacteria into lactic acid and acetic acid, increasing intestinal osmotic pressure, retaining water, and stimulating intestinal motility, leading to diarrhea. Bacterial fermentation of lactulose produces gas (e.g., hydrogen, carbon dioxide), causing bloating and intestinal cramps. Long-term use may disrupt gut microbiota balance, worsening gastrointestinal symptoms. Patients with cardiac insufficiency, the elderly, or those with renal impairment should use these drugs with caution, and electrolytes (especially potassium and sodium) and circulatory volume should be monitored regularly. These adverse reactions were transient and did not require discontinuation of the medication. By reducing the dosage or slowing the infusion rate, the aforementioned adverse reactions could resolve. However, further research and discussion are still needed.

## 5 Limitations

This article acknowledges certain limitations: the majority of the studies included in the meta-analysis did not report on blinding methods, which may introduce a risk of bias in the study results. Due to the limitations in the quantity and quality of the included studies, the above conclusions await further validation from larger-sample, high-quality, and multicenter studies. We will discuss the combination therapy in the future compared to lactulose alone and LOLA alone.

## 6 Conclusion

Based on existing research data, we analyzed the efficacy and safety of LOLA and lactulose, and provided comprehensive information on patient clinical characteristics. The meta-analysis results indicated that the combination of LOLA and lactulose in the treatment of hepatic encephalopathy has a higher total effectiveness in clinical practice and can significantly reduce indicators such as AST, ALT, TBIL, and NH3. In terms of safety, it also suggests that the incidence of adverse reactions with combined treatment is lower and the safety is higher. Therefore, the conclusion is drawn that LOLA combined with lactulose significantly reduces neurotoxins, improves liver function recovery, and enhances therapeutic effects.

This study provides a certain reference for the safe use of LOLA and lactulose in clinical practice. LOLA has clinical practical value in the treatment of patients with hepatic encephalopathy and is worth further promotion and use. Future research should explore the potential mechanisms related to LOLA and lactulose.

## Data Availability

The original contributions presented in the study are included in the article/Supplementary material, further inquiries can be directed to the corresponding author.
