# Supplementary material for: Efficacy and safety of L-ornithine L-aspartate combined with lactulose in treatment of hepatic encephalopathy: a systematic review and meta-analysis of randomized controlled trial
Source: Front Med (Lausanne). 2025 Apr 30;12:1581792. doi: 10.3389/fmed.2025.1581792 (PMC12075176; doi:10.3389/fmed.2025.1581792)
Supplement: Supplementary file 1 [file Data_Sheet_1.docx]

Supplementary Material 1

| Order | Strategy |
| --- | --- |
| #1 | Search: Hepatic Encephalopathy[MeSH Terms] |
| #2 | Search: "encephalopathies hepatic"[Title/Abstract] OR "hepatic encephalopathies"[Title/Abstract] OR "portosystemic encephalopathy"[Title/Abstract] OR "portosystemic encephalopathies"[Title/Abstract] OR "encephalopathy hepatic"[Title/Abstract] OR "encephalopathy hepatocerebral"[Title/Abstract] OR "Encephalopathies"[Title/Abstract]) OR "encephalopathy portal systemic"[Title/Abstract] OR "encephalopathy portal systemic"[Title/Abstract] OR "portal systemic encephalopathies"[Title/Abstract] OR "encephalopathy portosystemic"[Title/Abstract] OR "hepatocerebral encephalopathy"[Title/Abstract] OR "portal systemic encephalopathy"[Title/Abstract] |
| #3 | Search: #1 OR #2 |
| #4 | Search: "ornithylaspartate"[MeSH Terms] |
| #5 | Search: "Orn-Asp"[Title/Abstract] OR "ornithine aspartate"[Title/Abstract] OR "gepa-merz"[Title/Abstract] OR "L-ornithine-L-aspartate"[Title/Abstract] |
| #6 | Search: #4 OR #5 |
| #7 | Search: "randomized controlled trial"[Publication Type] OR "Randomized"[Title/Abstract] OR "Placebo"[Title/Abstract] |
| #8 | Search: #3 AND #6 AND #7 |
